# Pulse Design of Constant Strain Rate Loading in SHPB Based on Pulse Shaping Technique

**DOI:** 10.3390/ma17122931

**Published:** 2024-06-14

**Authors:** Shengpeng Chen, Runqiang Chi, Wuxiong Cao, Baojun Pang, Zhenlong Chao, Longtao Jiang, Tian Luo, Runwei Zhang

**Affiliations:** 1 School of Astronautics, Harbin Institute of Technology, Harbin 150001, China; shengpeng.chen@hit.edu.cn (S.C.); chirq@hit.edu.cn (R.C.); pangbj@hit.edu.cn (B.P.); 2 School of Materials Science and Engineering, Harbin Institute of Technology, Harbin 150001, China; longtaojiang@163.com (L.J.); luotian1452@gmail.com (T.L.); zrw920127@163.com (R.Z.)

**Keywords:** pulse-shaping technique, constant strain rate (CSR), B_4_C_P_/Al composites, pulse design

## Abstract

The Split Hopkinson pressure bar (SHPB) is widely used for characterizing the mechanical behavior of materials at high strain rates. One of the most challenging factors is achieving constant strain rate (CSR) loading of the specimen at a certain strain rate. Obtaining the effective incident pulse based on the experimental material for achieving CSR loading remains unresolved. This research focuses on obtaining the proper incident pulse for achieving constant strain rate loading using the pulse-shaping technique. A parameterized objective incident model in terms of the strain rate and quasi-static (or dynamic stress–strain) behavior of the material is established utilizing the three-wave method. Experimental pulses that closely resemble the desired objective pulses can be generated by adjusting parameters such as the geometry of the shaper, the shaper material, striker velocities, and the length of the striker according to the pulse-shaping model. The model is applied to the design of the incident pulse for B_4_C_P_/2024Al composite material, and the dynamic stress–strain curves at different strain rates are obtained under CSRs. This model provides effective guidance for selecting an appropriate shaper and achieving CSR loading in SHPB tests.

## 1. Introduction

The Split Hopkinson pressure bar (SHPB) is widely used for characterizing the mechanical behavior of materials at high strain rates, typically in the range of 10^2^ to 10^4^ s^−1^ [1,2]. This technique was improved by Kolsky [3,4]. However, the trapezoid incident pulse generated by the traditional SHPB (without a shaper) is only suitable for testing low work-hardening materials, such as copper and aluminum alloys [5,6,7]. It is not applicable to test the dynamic behavior of materials with considerable work-hardening [8,9] or brittle materials such as ceramics [10] and rocks [11], which have near-linear stress–strain relationships. Moreover, there has been widely growing interest in a variety of materials’ dynamic mechanical behavior with complex stress–strain behavior in recent years, such as Hydrogels by Harnessing 2D Hierarchical Structures [12], metal matrix composites [13,14], biomaterials [15,16], and high-entropy alloys [17]. Thus, various pulse-shaping methods have been proposed to meet the loading conditions. These loading conditions require CSR [8,18], stress uniformity [5], and elimination of dispersion et al. [19,20]. One of the most challenging problems is to achieve CSR loading of the specimen at different strain rates.

Various methods have been developed for SHPB (or SHTB) tests, such as shaping the striker, utilizing a preloading bar, and employing a pulse shaper to tailor the incident pulse shape to meet the dynamic loading conditions. Duffy et al. [21] first proposed the pulse-shaping technique in the Split Hopkinson Tension Bar (SHTB) test to achieve a smooth incident pulse waveform generated by the explosion. Compared to the trapezoid rising edge of the incident pulse, smooth rising edge pulses are more favorable for eliminating oscillations and obtaining CRS loading. Li et al. designed a striker bar with a truncated cone on both ends to produce a half-sine incident pulse for testing rock materials [22,23]. The half-sine incident pulse eliminated oscillations and provided a better stability strain rate loading in the specimens. Parry et al. placed a preloading bar with a strength lower than that of the Hopkinson bars between the striker and the input bar to reduce the dispersion effect [19]. Utilizing front and rear pulse shapers, Song et al. developed a pulse-shaping technique for both loading and unloading paths of SHPB experiments for nickel-titanium shape-memory alloys to achieve dynamic stress equilibrium and nearly CSR for specimens in both elastic and plastic regions [24]. Vecchio et al. selected a kind of material with high strength and a high work-hardening rate to fabricate pulse shapers and tested several specimens with a wide range of strength and work-hardening behavior [8]. They determined the proper dimensions of the pulse shaper for different test conditions via experimental trials. Li et al. [25] discussed the pulse-shaper diameter in the SHPB test and indicated that a shaper with a certain diameter can only offer one optimum near CSR. Therefore, using a pulse shaper to tailor the incident pulse is the main method of pulse shaping.

Modeling the plastic deformation of the pulse shaper has been considered by Nemat-Nasser et al. [26]. They used a power-law relation in a one-dimensional wave propagation analysis to predict the incident pulse for OFHC copper (Oxygen-Free Hard Copper) pulse shapers. Frew et al. [10,27,28] extended the model proposed by Nasser et al. [26] to obtain a high strain rate and stress–strain data for brittle and elastic–plastic materials. They discussed the effects of the size of the pulse shaper, the length, and the velocity of the striker bar on incident pulse waveforms based on the model. Pang et al. [29] improved the theoretical model [10] to analyze the effects of shaper density and geometry parameters on the incident pulse. Using commercial software (LS-DYNA V971) to guide the selection of a shaper is also an acceptable method. Ramirez et al. [30] studied the effects of pulse shaper materials and dimensions on the incident pulse. Naghdabadi et al. [20] studied the effect of pulse shaper thickness, pulse shaper diameter, striker bar length, and striker bar velocity on the incident pulse shape via simulations. However, the incident pulse at a specific strain rate for the material under study has only been evaluated empirically. Based on this evaluation, most researchers adjust the experimental parameters of the pulse shaper to achieve the desired loading pulse. This adjustment process necessitates a significant number of experimental trials.

In this study, a model is proposed to determine the objective incident pulse based on the quasi-static or dynamic stress–strain curves of the material under investigation. The rising edges of incident pulses produced by a ductile shaper (copper) and a soft-material shaper (rubber) are compared under the conditions of stress uniformity. We obtain objective incident pulses for the three typical ideal materials at different strain rates and briefly discuss the influences of the different kinds of quasi-static stress–strain curves on the loading pulse. In addition, the objective incident pulse model proposed in this paper is validated by the experiments.

## 2. Method for Objective Incident Pulse at CSR

### 2.1. Objective Incident Pulse Model

Song et al. [31,32] demonstrated that the strain rate of a specimen undergoing uniform plastic deformation can be determined using the three-wave method, as expressed in the following equation:

(1) $${\dot{\varepsilon }}_{s}=\frac{c_{o}}{L_{s}\left(t\right)}\left[\varepsilon_{i}\left(t\right)-\varepsilon_{r}\left(t\right)-\varepsilon_{t}\left(t\right)\right]$$

**Figure 1:**
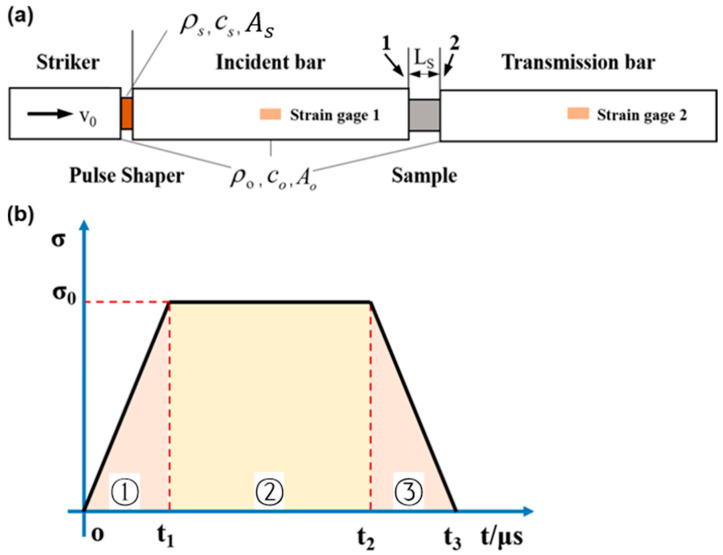
(**a**) Schematic of a Split Hopkinson pressure bar with the pulse shaper; (**b**) incident pulse without pulse shaper, the linear ramp loading region (➀), the constant stress loading region (➁), and the linear ramp unloading region (➂).

The average flow stress of the specimen can be obtained by [31]

(2) $${\bar{\sigma }}_{12}\left(t\right)=\frac{A_{o}}{2A_{s}\left(t\right)}E_{o}\left[\varepsilon_{i}\left(t\right)+\varepsilon_{r}\left(t\right)+\varepsilon_{t}\left(t\right)\right]$$

where ${\dot{\varepsilon }}_{s}$ and ${\bar{\sigma }}_{12}\left(t\right)$ are the strain rate and the average flow stress of the specimen. $L_{s}\left(t\right)$ and $A_{s}\left(t\right)$ are the length and cross-sectional area of the specimen at time t, respectively. $A_{o}$, $c_{o}$ and $E_{o}$ are the cross-sectional area, one-dimensional elastic wave speed, and elastic modulus of the bar, respectively. $\varepsilon_{i}\left(t\right)$ and $\varepsilon_{r}\left(t\right)$ are incident and reflected strain measured by Strain gage 1 mounted on the incident bar. $\varepsilon_{t}\left(t\right)$ is transmitted strain measured by Strain gage 2 mounted on the transmission bar as shown in Figure 1. The two equations above are transformed into stress form.

(3) $$\rho_{o}c_{o}L_{s}\left(t\right){\dot{\varepsilon }}_{s}=\sigma_{i}\left(t\right)-\sigma_{r}\left(t\right)-\sigma_{t}\left(t\right)$$

(4) $$\frac{2A_{s}\left(t\right)}{A_{o}}{\bar{\sigma }}_{12}\left(t\right)=\sigma_{i}\left(t\right)+\sigma_{r}\left(t\right)+\sigma_{t}\left(t\right)$$

Here, $\rho_{o}$ is the bar density. $\sigma_{i}\left(t\right)$, $\sigma_{r}\left(t\right)$, and $\sigma_{t}\left(t\right)$ are incident, reflected, and transmitted stress. Then the incident stress $\sigma_{i}\left(t\right)$ can be written as

(5) $$\sigma_{i}\left(t\right)=\frac{A_{s}\left(t\right)}{A_{o}}{\bar{\sigma }}_{12}\left(t\right)+\frac{1}{2}\rho_{o}c_{o}L_{s}\left(t\right){\dot{\varepsilon }}_{s}$$

The strain rate ${\dot{\varepsilon }}_{s}$ of the specimen is a constant value during the loading, and the length of the specimen can be written as

(6) $$L_{s}\left(t\right)=L_{s}(1-{\dot{\varepsilon }}_{s}t)$$

Given that most of the materials are incompressible and experience homogeneous deformation during the testing [10,31], we have

(7) $$A_{s}\left(t\right)=\frac{A_{s}}{1-{\dot{\varepsilon }}_{s}t}$$

Then the incident loading $\sigma_{i}\left(t\right)$ can be written as

(8) $$\sigma_{i}\left(t\right)=\frac{A_{s}}{A_{o}\left(1-{\dot{\varepsilon }}_{s}t\right)}{\bar{\sigma }}_{12}\left(t\right)+\frac{1}{2}\rho_{o}c_{o}L_{s}\left(1-{\dot{\varepsilon }}_{s}t\right){\dot{\varepsilon }}_{s}$$

where $A_{s}$ and $L_{s}$ represent the initial cross-sectional area and length of the specimen, respectively.

The quasi-static stress–strain curve of the specimen, denoted as σ-ε, is generally obtained by measurement. The dynamic stress–strain curve of the material exhibits a consistent shape and trend with the quasi-static stress–strain curve at lower loading strain rates [33]. Moreover, obtaining a stable and reliable quasi-static stress–strain curve is easier compared to the dynamic one. Therefore, the quasi-static stress–strain curve of the material can be used to design the incident pulse waveform in the initial stages. We consider the reliable quasi-static stress–strain curve as follows:

(9) $$\sigma =f\left(\varepsilon \left(t\right)\right)$$

Then the flow stress $\sigma_{y}$ and plastic strain $\varepsilon_{p}$ can be obtained by removing the elastic response of Equation (9):

(10) $$\sigma_{y}\left(\varepsilon_{p}\right)=g\left(\varepsilon_{p}\left(t\right)\right)$$

Assuming that the stress–strain curve $\sigma_{y}\left(\varepsilon_{p}\right)$ of the material is determined at the strain rate ${\dot{\varepsilon }}_{s}$, the flow stress–strain curve at the loading strain rate ${\dot{\varepsilon }}_{s}$ can be written as

(11) $$g\left(\varepsilon_{p}\left(t\right)\right)=g\left({\dot{\varepsilon }}_{s}t\right)$$

The flow stress–strain curve $\sigma_{y}\left(\varepsilon_{p}\right)$ of Equation (11) is substituted into Equation (8). Then the incident stress at the strain rate ${\dot{\varepsilon }}_{s}$ becomes

(12) $$\sigma_{i}\left(t\right)=\frac{A_{s}}{A_{o}\left(1-{\dot{\varepsilon }}_{s}t\right)}g\left({\dot{\varepsilon }}_{s}t\right)+\frac{1}{2}\rho_{o}c_{o}L_{s}\left(1-{\dot{\varepsilon }}_{s}t\right){\dot{\varepsilon }}_{s}$$

When the time t=0, the yield stress $\sigma_{Y}$ of the material equals $g\left(0\right)$. The velocity $V_{0}$ of the striker can be predicted by

(13) $$V_{0}\left(A_{s},\sigma_{Y},{\dot{\varepsilon }}_{s}\right)=\frac{2A_{s}}{A_{o}\rho_{o}c_{o}}\sigma_{Y}+L_{s}{\dot{\varepsilon }}_{s}$$

As the strain rate of the test increases, the strain rate effect of the material becomes more pronounced. The quasi-static stress–strain curve of the material shows a clear difference compared to the stress–strain curve under the high-strain-rate conditions. Therefore, the loading pulse also needs to be adjusted accordingly. It is assumed that the stress–strain curve $\sigma_{y}^{i}\left(\varepsilon_{p}\right)$ of the material at the strain rate ${\dot{\varepsilon }}_{i}$ is determined from the above SHPB tests. The prediction incident pulse at the strain rate ${\dot{\varepsilon }}_{i}+\Delta \dot{\varepsilon }$ becomes

(14) $$\sigma_{i}^{{\dot{\varepsilon }}_{i}+\Delta \dot{\varepsilon }}\left(t\right)=\frac{A_{s}}{A_{o}\left(1-({\dot{\varepsilon }}_{i}+\Delta \dot{\varepsilon })t\right)}g\left(({\dot{\varepsilon }}_{i}+\Delta \dot{\varepsilon })t\right)+\frac{1}{2}\rho_{o}c_{o}L_{s}\left(1-({\dot{\varepsilon }}_{i}+\Delta \dot{\varepsilon })t\right)({\dot{\varepsilon }}_{i}+\Delta \dot{\varepsilon })$$

The velocity $V_{0}$ of the striker can be predicted by

(15) $$V_{0}\left(A_{s},\sigma_{Y}^{{\dot{\varepsilon }}_{i}},{\dot{\varepsilon }}_{i}+\Delta \dot{\varepsilon }\right)=\frac{2A_{s}}{A_{o}\rho_{o}c_{o}}\sigma_{Y}^{{\dot{\varepsilon }}_{i}}+L_{s}({\dot{\varepsilon }}_{i}+\Delta \dot{\varepsilon })$$

### 2.2. Analysis of Stress Uniformity

Equation (16) provides a method for calculating the plastic strain of the shaper and the stress transmitted to the incident bar [34]. Considering the incident pulse with a linear ramp (positive in compression) [5,10,29], the typical pulse, without using the pulse shaper, is given by Equation (16c). The one-dimensional resistance for copper in Equation (17a) was provided by Lu [34], while the function of rubber in Equation (17b) was derived by fitting the stress–strain data obtained from SHPB tests. M is the stress loading rate, $t_{1}$ is the end of the linear ramp, $t_{2}$ is the starting time of unloading and $t_{2}=2L_{o}/c_{o}$ that is determined by the length and elastic speed of the striker [35]. $t_{3}$ represents the end time of the loading, and $c_{o}$ is the one-dimensional elastic wave speed of the bar. Figure 1b portrays the incident pulse calculated by Equation (16c), and the stress loading rate M can be determined by $M=\sigma_{0}/t_{1}$. The incident pulse without a shaper depicted in Figure 1b consists of three regions: the linear ramp loading region (➀), the constant stress loading region (➁), and the linear ramp unloading region (➂).

Two typical pulse-shaping materials, H62 copper and rubber, were selected for this study as they are usually used in SHPB experiments [34]. The basic parameters of the pulse-shaping materials and the impact velocities are given in Table 1. The striker bar and the incident bar are made of the same linear elastic spring steel shown in Figure 1. Young’s modulus is 200 GPa, the density is 7800 kg/m^3^, and the diameter is 12.7 mm. The length of the striker bar is 300 mm, and its initial velocity changes with the pressure of the pressure chamber. The equation for predicting the incident stress can be written as

**Figure 2:**
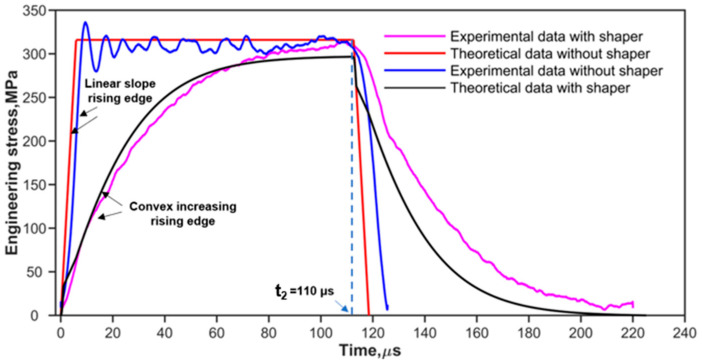
Experimental and theoretical incident waves at impact velocity of 16 m/s.

**Figure 3:**
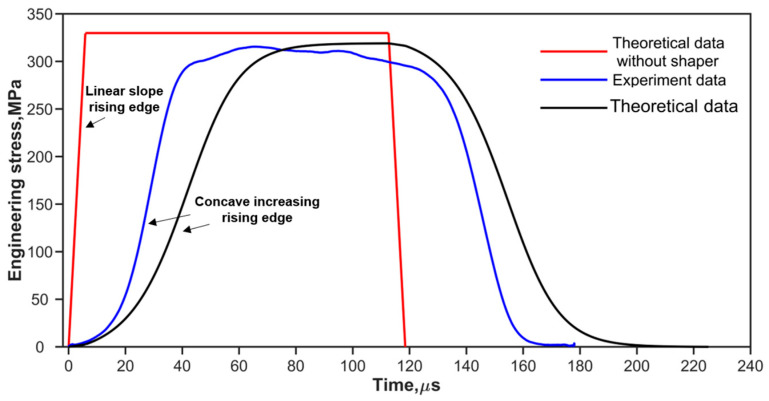
Experimental and theoretical incident waves at impact velocity of 16.7 m/s.

(16) ε˙pt=V0hs−2fs(εpt)asAoρocohs1−εpt (a)σit=fs(εpt)asAo1−εpt0≤t<t2(b)or  σit=MtMt10≤t<t20≤t<t2(c)

(17) $$\left\{\begin{array}{ll} f_{s}^{copper}=\frac{650\varepsilon_{p}^{0.12}}{1-\varepsilon_{p}^{4.5}} & \left(a\right)\\ f_{s}^{rubber}=106.5e^{3.646\varepsilon_{p}} & \left(b\right) \end{array}\right.$$

where $f_{s}(\varepsilon_{p}\left(t\right))$ is the one-dimensional resistance function [10,28] of the shaper. $a_{s}$ and $h_{s}$ are the original cross-sectional area and thickness of the pulse shaper, respectively. ${\dot{\varepsilon }}_{p}\left(t\right)$ is the strain rate of the pulse shaper. $A_{o}$, $\rho_{o}$, and $c_{o}$ are the same as described in Section 2.1. $V_{0}$ is the impact velocity of the striker bar. Figure 2 and Figure 3 show the predicted (theoretical) and experimental results, respectively, for the cases of shaping and non-shaping. The stress wave begins to unload after $t_{2}$. The stress wave follows the elastic wave unloading law during the unloading. The experimental pulses obtained after shaping are in agreement with the theoretical pulse calculated by Equations (16) and (17).

The stress uniformity during the SHPB test can be measured by [36]

(18) $$R\left(t\right)=\frac{2\left(\sigma_{1}-\sigma_{2}\right)}{\sigma_{1}+\sigma_{2}}$$

Generally, when $R\left(t\right)<5\%$, it indicates that the stress has reached a state of uniformity. Interface stresses $\sigma_{1}$ and $\sigma_{1}$ can be derived from the stress wave propagation principle between specimen and bar [35,37]. The stress subjected to the bar of interface 1 in Figure 1a is $\sigma_{1}$

$$\sigma_{1}\left(t\right)=\left\{\begin{array}{l} M\left(1+F_{o-s}\right)t\;\;\;0\leq t<2t_{0}\\ \begin{array}{l} M\left(1+F_{o-s}\right)t+T_{o-s}F_{s-o}M\left(t-2t_{0}\right)T_{s-o}\\ +\cdots +T_{o-s}F_{s-o}^{2k-1}M\left(t-2kt_{0}\right)T_{s-o}\;2kt_{0}\leq t<2(k+1)t_{0} \end{array} \end{array}\right.$$

where k=1, 2, 3, ⋯. The stress subjected to the bar of interface 2 is $\sigma_{2}$

$$\sigma_{2}\left(t\right)=\left\{\begin{array}{ll} 0 & 0\leq t<t_{0}\\ T_{o-s}M\left(t-t_{0}\right)T_{s-o}+T_{o-s}F_{s-o}^{2}M\left(t-3t_{0}\right)T_{s-o}\\ +\cdots +T_{o-s}F_{s-o}^{2\left(k-2\right)}M\left(t-\left(2k-3\right)t_{0}\right)T_{s-o} & \left(2k-3\right)t_{0}\leq t<\left(2k-1\right)t_{0} \end{array}\right.$$

**Figure 4:**
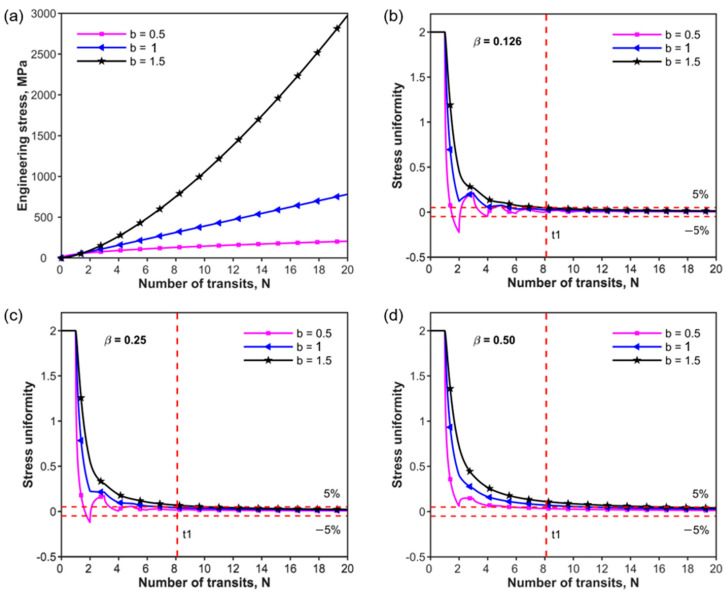
Variation in stress uniformity with number of wave transits for different mechanical impedance ratios β of power law incident loading: (**a**) power law incident loading rising edge with parameters b=0.5, 1, and 1.5; (**b**) stress uniformity at β=0.126 of power law incident loading rising edge; (**c**) stress uniformity at β=0.25 of power law incident loading; (**d**) stress uniformity at β=0.5 of power law incident loading rising edge.

The experimental pulses shown in Figure 2 and Figure 3 indicate that convex and concave increasing rising edges can be achieved by using ductile metal (copper) and soft (rubber) shapers, respectively. To simplify the analysis of the effect of the rising edge of the pulse on stress uniformity, the rising edges of loading waveforms are simplified to the power law [38] as shown in Equation (19).

(19) $$\sigma_{i}\left(t\right)=Mt^{b}$$

where b is a parameter that defines the variation of the loading curve and b=1 represents the case of the linear slope rising edge of the loading curve. b<1 and b>1 represent loading curves with convex and concave increasing edges, respectively. Two specific-power low-incident curves with b=0.5 and b=1.5 are considered as shown in Figure 4a. Figure 4b–d shows the variations in stress uniformity over time for the three rising edges, plotted using Equations (18) and (19). The loading wave needs to transit N times from one end of the specimen to the other. The value of N is given by

(20) $$N=\frac{t{c^{\prime }}_{s}}{L_{s}}$$

where t is the time of the rising edge of the incident pulse, and ${c^{\prime }}_{s}$ and $L_{s}$ are the elastic wave speed and length of the specimen.

The relative mechanical impedance also has a significant effect on the stress uniformity. Figure 4b–d represents the stress uniformity of the three incident waves imposed on the three different samples with the relative mechanical impedance of β = 0.126, 0.25 and 0.5, respectively. β is the ratio between the mechanical impedances of the specimen and the input/output bar. According to Equation (18), a smaller value corresponds to a more uniform stress state. It can be observed that the transits N for stress uniformity increase with the increase in the value of the parameter b at the same mechanical impedance ratio β. Table 2 shows examples of how N varies with the relative mechanical impedance β and parameter b of the power law.

A comparison of the values in Table 2 (or Figure 4b–d) shows that the achievement of stress uniformity in a specimen for the rising edge with the parameter b=0.5 occurs earlier than that for the other two rising edges. For instance, stress uniformity requires eleven and fourteen wave transits for the power law incident pulse with parameter b=1 and 1.5 at β=0.5, respectively. However, only four transits are required for the power law rising edge with the parameter b=0.5 at β=0.5. Obviously, the effect of the rising edge of the incident pulse on stress uniformity should not be neglected during the SHPB experiments compared with relative mechanical impedance β.

### 2.3. Conditions for Constant Strain Rate (CSR) Experiment

The commonly used form for the dynamic constitutive behavior of metals at room temperature [39] is:

(21) $$\sigma =\sigma \left(\varepsilon ,\dot{\varepsilon }\right)$$

**Figure 5:**
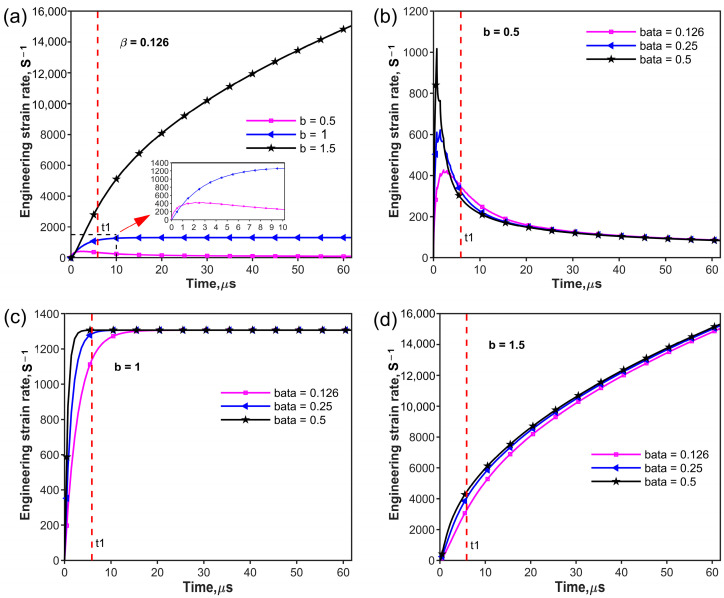
Variation in engineering strain rate with time: (**a**) Engineering strain rate of power law incident waves with β=0.126; (**b**) engineering strain rate at different beta values (β) with b=0.5 power law incident wave; (**c**) engineering strain rate at different beta values (β) with b=1 power law incident wave; (**d**) engineering strain rate at different beta values (β) with b=1.5 power law incident wave. The red dotted line indicates the end of the rising edge time $t_{1}$ of the pulse. The area enclosed by the black dotted line and the axes is shown enlarged where the red arrow point.

This constitutive equation was obtained by fitting experimental data, assuming constant strain rates, which introduces inevitable errors. The specimen is loaded by SHPB through a stage of rapid strain rate increase, after which it is maintained at a constant strain rate. In this section, the strain rate variation characteristics of the three kinds of incident pulse rising edges in Equation (19) were investigated. The strain rate of the specimen during the rising edge can be obtained by the following equation:

(22) $$\bar{\dot{\varepsilon }}=\left\{\begin{array}{l} \frac{T_{B-S}A_{o}\sigma_{i}\left(t\right)}{\rho_{ss}c_{ss}A_{s}h_{ss}}\;\;\;0\leq t<t_{0}\\ \frac{T_{B-S}A_{o}}{\rho_{ss}c_{ss}A_{s}h_{ss}}\left[\sigma_{i}\left(t\right)-\left(1-F_{S-B}\right)\sigma_{i}\left(t-t_{0}\right)-\cdots -\left(F_{S-B}^{k-1}+F_{S-B}^{k}\right)\sigma_{i}\left(t-kt_{0}\right)\right]\;kt_{0}\leq t<(k+1)t_{0} \end{array}\right.$$

where $\bar{\dot{\varepsilon }}$ is the average strain rate, $\sigma_{i}\left(t\right)$ is the rising edge of the incident pulse in Equation (19), $T_{B-S}$ is the wave transmission factor from the bar to the specimen, and $F_{S-B}$ is the wave reflection factor from the specimen to the bar. $A_{o}$ and $A_{ss}$ are the cross-sectional areas of the bar and the specimen, respectively. $\rho_{ss}$, $c_{ss}$, and $h_{ss}$ are the density, wave speed, and length of the specimen, respectively. k constitutes the number of wave transits. Figure 5 is plotted by Equation (22), which describes the variation in engineering strain rates over time. Figure 5a shows that the rising edge of the loading curve significantly affects the engineering strain rate curve. The power function with the parameter b=1.5 subjected to the specimen exhibits a continuous strain rate increase. The linear ramp loading curve led to a CSR after a rapidly rising strain rate. However, the power function with parameter b=0.5 brings about a continuous strain rate decrease after a sharp rise in strain rate. The relative mechanical impedance β does affect strain rate variation with time at the early loading stage shown in Figure 5b–d. Figure 5b shows that the maximum strain rate increases as the relative mechanical impedance β increases, and then the strain rate decreases to the same level. Figure 5c shows that smaller relative mechanical impedance will clearly extend the strain rate’s increase time, and the same results were concluded by Song [31]. For the concave increasing rising edge shown in Figure 5d, the similar increase curve of the strain rate with different relative mechanical impedance values indicates that the effect of relative mechanical impedance is negligible. This indicates that a certain rising edge of the incident curve determines a mode of strain rate–time variation. However, based on the analysis of stress uniformity in Section 2.2, it can be concluded that the convex increasing (b = 0.5) power law incident pulse exhibits favorable characteristics in terms of stress uniformity.

**Figure 6:**
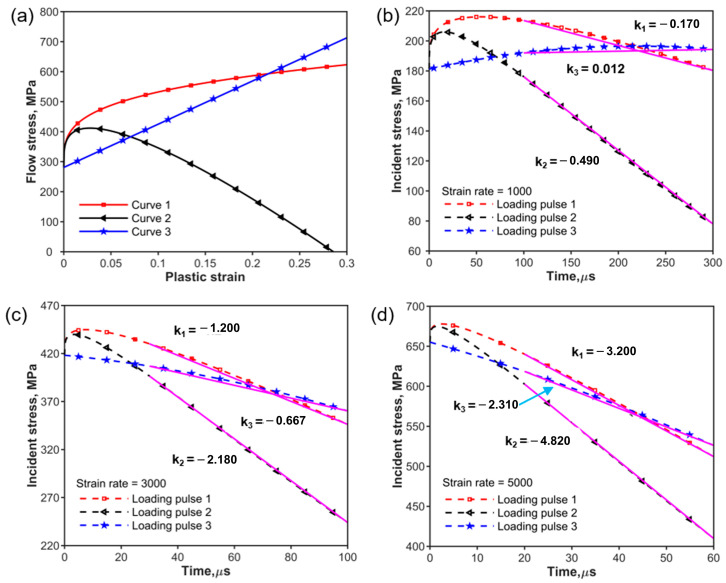
Variation in incident stress with time: (**a**) flow stress and plastic strain curves; (**b**) incident waveforms at strain rate = 1000 s^−1^ of the three curves; (**c**) incident waveforms at strain rate = 3000 s^−1^ of the three curves; (**d**) incident waveforms at strain rate = 5000 s^−1^ of the three curves and the slope K_3_ of Loading pulse 3 as blue arrow point.

The strain rate increases during the rising edge phase of the incident pulse. Once the test material begins to yield, it enters the phase of constant strain rate loading. To analyze the objective incident pulse required for materials with quasi-static stress–strain curves of different strain hardening levels, three types of quasi-static stress–strain curves were chosen to study the shape of the incident pulse at different strain rates. Figure 6a shows the three different stress–strain curves. Curve 1 represents the typical work-hardening stress–strain relationship as Equation (23) with the increase in the power law. Curve 2 in Figure 6a shows the simplified strain-softening material’s constitutive relationship obtained from Equation (24) and curve 3 represents the bilinear elastoplastic model written as Equation (25).

(23) $$\sigma_{y}\left(\varepsilon_{p}\right)=a+b\varepsilon_{p}^{n1}$$

(24) $$\sigma_{y}\left(\varepsilon_{p}\right)=\left(a+b\varepsilon_{p}^{n1}\right)\left(1-c\varepsilon_{p}^{m}\right)$$

(25) $$\sigma_{y}\left(\varepsilon_{p}\right)=\left(a+b\varepsilon_{p}\right)$$

where a, b, c, and m are constant, $\sigma_{y}$ is the flow stress, and $\varepsilon_{p}$ is the plastic strain. To facilitate comparison, we use the parameters (Table 3) to calculate the CSR objective incident pulse shown in Figure 6b–d. The objective incident waves are calculated by Equation (12) shown in Figure 6b–d at strain rates of 1000 s^−1^, 3000 s^−1^, and 5000 s^−1^. It can be seen that the trend of the objective incident stress with time is consistent with the trend of the corresponding stress–time curve in a quasi-static state at the beginning of the plastic deformation of the specimen. The incident stress decreases approximately linearly with time after a short rise for curve 1 and curve 2. At the lower strain rates (less than or equal to 1000 s^−1^) shown in Figure 6b, the shape of the objective incident pulse for constant strain rate loading is closer to the material stress–time curve of the quasi-static state in the initial loading stage. The loading stress rate decreases as the loading strain rate increases, as shown in Table 4 and Figure 6c,d. The loading stress rate is negative except for curve 3 where the loading stress rate is a small positive value (0.012) at a strain rate of 1000 s^−1^. This means that dynamic stress–strain curves at CSR can also be obtained for curves 1 and 3 (work-hardening material) using constant stress with a negative loading stress rate during the plastic deformation of the specimen. However, for curve 2 (strain-softening material), a loading pulse similar to a sinusoidal shape is used to obtain a stress–strain curve with sufficient accuracy at a CSR.

### 2.4. SHPB CSR Test for B_4_C_P_/2024Al Composites

**Figure 7:**
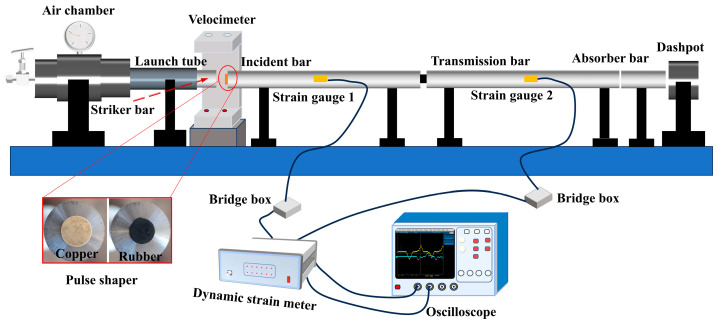
SHPB experimental equipment and setup.

**Figure 8:**
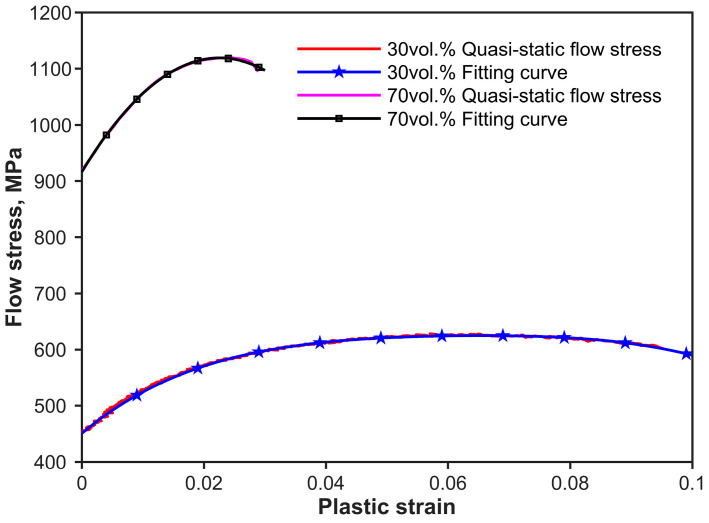
Quasi-static flow stress vs. plastic strain and corresponding fitting curves for 30 vol.% and 70 vol.% B_4_C_p_/2024Al composite materials.

Figure 7 shows the SHPB experimental equipment and setup. The striker bar and the incident bar are made of the same linear elastic spring steel described in Section 2.2. The impact velocity of the striker $V_{0}$ changes with the pressure of the pressure chamber. The input and output bars have the same length of 1200 mm. Experimental and theoretical striker velocities and the corresponding strain rates of composite specimen SHPB experiments are shown in Table 5. The basic parameters of the shaper materials are shown in Table 1. The strain rate is dominated by the velocity of the striker using Equation (13) or Equation (15). At the beginning of the test, the flow stress–strain curve of the quasi-static state in Figure 8 is substituted into Equation (12).

**Figure 9:**
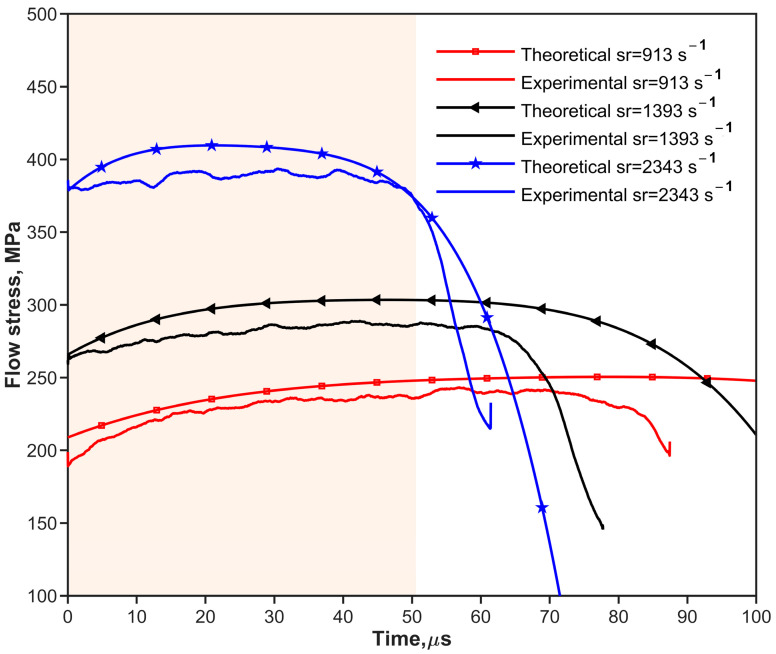
Theoretical and experimental loading stress vs. time for 30 vol.% B_4_C_p_/2024Al composite material at different strain rates. The orange area means the duration of the initial stage, during which the experimental and theoretical curves match well.

**Figure 10:**
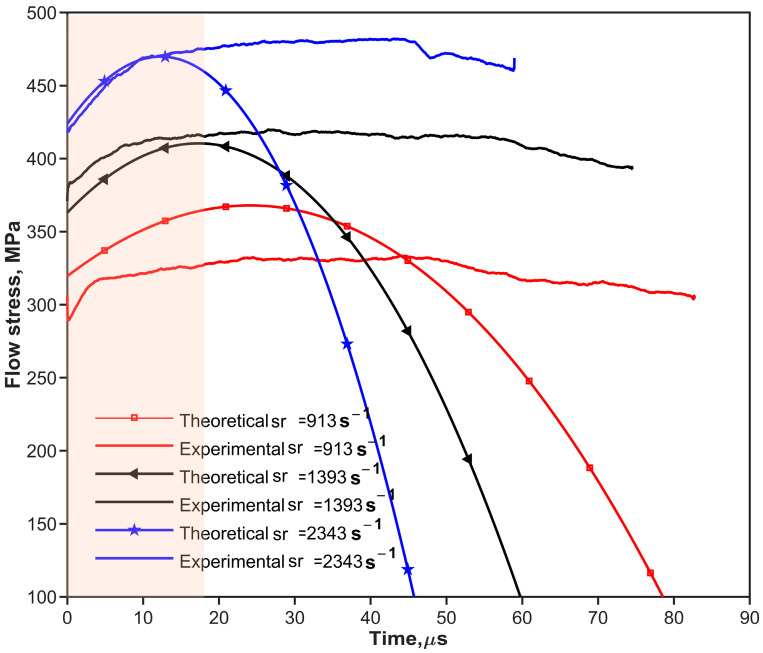
Theoretical and experimental loading stress vs. time for 70 vol.% B_4_C_p_/2024Al composite material at different strain rates. The orange area means the duration of the initial stage, during which the experimental and theoretical curves match well.

**Figure 11:**
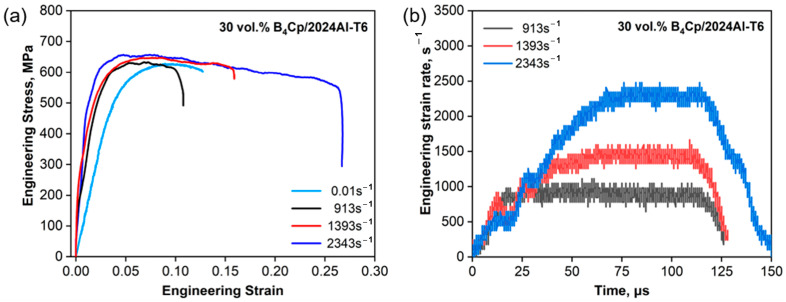
Engineering stress vs. engineering strain and strain rate vs. time of 30 vol.% B_4_C_p_/2024Al composite material under quasi-static and dynamic loads: (**a**) engineering stress vs. engineering strain of 30 vol.% B_4_C_p_/2024Al composite material subjected to quasi-static and dynamic loads; (**b**) strain rate vs. time of 30 vol.% B_4_C_p_/2024Al composite material subjected to dynamic load.

**Figure 12:**
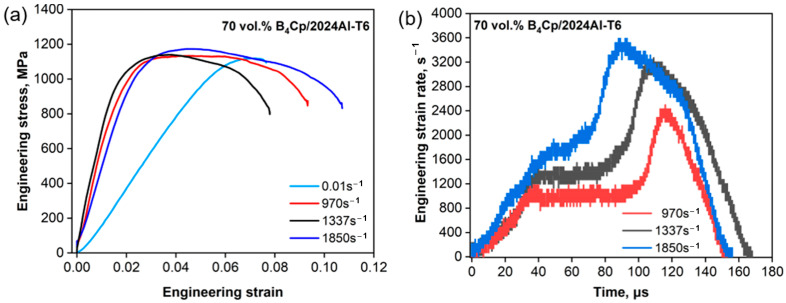
Engineering stress vs. engineering strain and strain rate vs. time of 70 vol.% B_4_C_p_/2024Al composite material under quasi-static and dynamic loads: (**a**) engineering stress vs. engineering strain of 70 vol.% B_4_C_p_/2024Al composite material subjected to quasi-static and dynamic loads; (**b**) strain rate vs. time of 70 vol.% B_4_C_p_/2024Al composite material subjected to dynamic load.

Figure 9 and Figure 10 show all the objective incident pulses (theoretical curves) of 30 vol.% and 70 vol.% B_4_C_P_/2024Al composites at the intended strain rates (Theo.Sr) in Table 5. The quasi-static stress–strain curves can be obtained by the experiments shown in Figure 11a and Figure 12a at a strain rate of 0.01 s^−1^. Equations (26) and (27) of the composites are the initial curves obtained by fitting the experimental points after the yield point at 2% residual plastic strain at a strain rate of 0.01 s^−1^. Both the polynomial equations are substituted into Equation (12) as $\sigma_{y}\left(\varepsilon_{p}\right)$, respectively. The objective loading curves (theoretical curves) in Figure 9 and Figure 10 are obtained. As can be seen in Figure 11a and Figure 12a, after the first step, the engineering stress–strain curves at 1000 s^−1^ of the two composites do not show a significant strain rate effect.

It takes more than 25 us before the strain rate (Figure 11b and Figure 12b) arrives at a stable strain rate value. The wave speed of the 30 vol.% and 70 vol.% B_4_C_P_/2024Al composites are shown in Table 6. The transits N can be calculated using Equation (20). The specimens for the 30 vol.% B_4_C_P_/2024Al composite reached stress uniformity as N = 42 > N = 14. Based on the data presented in Figure 12b, it is clear that the 70 vol.% B_4_C_P_/2024Al samples are given sufficient time to reach a state of uniform stress before the CSR achieves a steady value during testing. Thus, we can use the quasi-static curve as the input to obtain the response at higher strain rates.

(26) $$g_{30vol\%}\left(\varepsilon_{p}\right)=-7.564\times 10^{6}\varepsilon_{p}^{4}+1.860\times 10^{6}\varepsilon_{p}^{3}-1.868\times 10^{5}\varepsilon_{p}^{2}+9033\varepsilon_{p}+451$$

(27) $$g_{70vol\%}\left(\varepsilon_{p}\right)=-3.979\times 10^{5}\varepsilon_{p}^{2}+1.795\times 10^{4}\varepsilon_{p}+917$$

In Table 5, Sr is the simple form of the strain rates. The theoretical and experimental striker velocities are simplified as Theo.V and Exp.V, respectively. The intended strain rates for 30 vol.% B_4_C_P_/2024Al composite material are 913 s^−1^, 1393 s^−1^, and 2343 s^−1^ and the corresponding striker velocities are 11.10 m/s, 14.10 m/s, and 20.10 m/s. To prevent the dynamic Hopkinson bar from taking any damage, a maximum strain rate of 1850 s^−1^ has been established for the 70 vol.% B_4_C_P_/2024Al composite material. The other two intended strain rates for the 70 vol.% B_4_C_P_/2024Al composite material are nearly the same as the 30 vol.% B_4_C_P_/2024Al composite material. The corresponding theoretical velocities calculated by Equation (13) are shown in Table 5.

The traditional pulse-shaping model [10,26,29] was adopted to obtain the pulse in the incidence rod. According to the guidelines proposed by Naghdabadi et al. [20], a pulse shaper with the appropriate material and geometric dimensions is selected to meet the condition that the strain rate is constant. The filtering of the experimentally obtained pulses in Figure 9 and Figure 10 facilitates the comparison of the similarities and differences between the pulses obtained by the proposed model and the experimental pulses guided by the proposed model. According to Figure 9, maintaining a longer constant loading stress ensures a CSR for the ductile 30 vol.% B_4_C_P_/2024Al composite material. Material with better plasticity implies the need for a longer loading time, as was discussed [20]. A ductile metal shaper is the best choice. We selected a copper pulse shaper with Ø11 × 1 mm to perform SHPB experiments for 30 vol.% B_4_C_P_/2024Al composite material. The experimental results are shown in Figure 9 and Figure 11.

Figure 10 indicates that a soft pulse shaper could be useful to test brittle materials such as 70 vol.% B_4_C_P_/2024Al composite material. The incident pulse shaped by the soft shaper has a longer rising edge compared with the ductile metal shaper and provides a slightly decreased slope after the incident pulse reaches the maximum value. The shaper of this incident wave is close to the half-sinusoidal pulse. Compared with the 30 vol.% B_4_C_P_/2024Al composite material, the loading pulse of the 70 vol.% B_4_C_P_/2024Al composite material is shorter. We selected rubber as the pulse shaper for the 70 vol.% B_4_C_P_/2024Al composite material. The sizes of the rubber shaper for the tests are Ø4 × 1 mm and Ø4 × 1.5 mm. Figure 10 and Figure 12 show the results of the experiments.

The different strain rate engineering stress–strain curves in Figure 11a and Figure 12a are filtered to show the effect of the strain rate more clearly. The dynamic stress–strain curves of 30 vol.% B_4_C_P_/2024Al composite specimens at different strain rates are shown in Figure 11a. The dynamic stress–strain curves exhibit consistency in shape and trend with the quasi-static stress–strain curve. It is shown that the experimental data obtained with the copper shaper achieves CSR loading and satisfies the assumption of stress uniformity during loading for 30 vol.% B_4_C_P_/2024Al composite specimens. 

The dynamic stress–strain curves shown in Figure 11a and Figure 12a can be regarded as ideal stress–strain curves due to the fulfillment of the conditions required for the CSR SHPB experiment. Figure 9 and Figure 10 depict the incident pulse waveforms at the experimental strain rates corresponding to the theoretical strain rates. The experimental loading pulse aligns well with the theoretically calculated loading pulses, indicating good agreement between the two pulses shown in Figure 9 for 30 vol.% B_4_C_P_/2024Al composite. In comparison, for the 70 vol.% B_4_C_P_/2024Al composite, the experimental loading curve only matches the corresponding theoretical loading curve during the early stages of loading, as shown in Figure 10. However, in the later stages, there is a significant difference between them. Fortunately, the duration of the initial stage, during which the curves match well, is sufficiently long to obtain the stress–strain relationship for the 70 vol.% B_4_C_P_/2024Al composite under CSR conditions.

## 3. Conclusions

This paper presents an objective pulse model for CSR loading. The effect of the rising edge of the objective pulse on the stress uniformity and the strain rate variation characteristics for a specific specimen is analyzed. Then, for three typical quasi-static stress–strain curves with different strain-hardening types, the objective pulses that may be required are analyzed. Finally, to validate the model, the dynamic stress–strain curves of two-volume-fraction B_4_C_p_/2024Al composite materials at constant strain rates were obtained using this model. The model provides guidance for implementing CSR loading in SHPB tests. This paper reveals:

(1) The objective incident pulse model provides the pulse profile, which is a target for the pulse shaping. One of the obvious differences between the two pulses shaped by the ductile metal shaper (H62 Copper) and the soft shaper (rubber) is the rising edge.
(2) Stress uniformity during loading is influenced by both the relative mechanical impedance and the rising edge of the incident pulse. Different types of rising edges of the incident pulse do affect the stress uniformity. The time of uniformity increases with the increase in parameter b in the power function.
(3) The rising edge of the incident pulse has a large effect on strain rate history and determines the mode of strain rate–time variations. For the concave rising edge (b = 1.5), the effect of relative mechanical impedance on stress uniformity is negligible, and it is easier to meet the high strain rate loading requirements for the specimen, though the convex increasing (b = 0.5) power law rising edge exhibits favorable characteristics in terms of stress uniformity.
(4) For materials with low strain hardening and strain softening, a negative stress rate pulse facilitates the constant strain rate loading of specimens after experiencing the rising edge of the incident pulse.

## Figures and Tables

**Table 1:** Parameters of shapers and impact velocities.

Material	Density (g/cm^3^)	Radius (cm)	Young Module (GPa)	Yield Stress (MPa)	Poisson Ratio	Velocity (m/s)	Thickness (mm)
H62 Copper	8.43	0.70	131	112.0	0.31	16.0	1.00
Rubber	1.93	0.40	0.025	4.5	0.47	16.7	1.00

**Table 2:** Number of wave transits N equired for $R\left(t\right)\leq 5\%$ (stress uniformity) shown in Figure 4.

β (Relative Mechanical Impedance)	*b* (Parameter of Power Law)		
	0.5	1.0	1.5
0.126	3	4	8
0.250	3	7	9
0.500	4	11	14

**Table 3:** Parameters of the curves.

Curve	a (MPa)	b (MPa)	n1	c	m
1	281	480	0.281	-	-
2	281	480	0.281	3.5	1
3	281	1440	1	-	-

**Table 4:** Loading stress rate for curves at different strain rates in Figure 6.

Strain Rate (s^−1^)	Stress Rate, (MPa/us)		
	k_1_	k_2_	k_3_
1000	−0.170	−0.490	0.012
3000	−1.200	−2.180	−0.667
5000	−3.200	−4.820	−2.310

**Table 5:** Experimental and theoretical striker velocities and corresponding strain rates shown in Figure 8 and Figure 9.

B_4_C_p_/2024Al Composites	30 vol.%			70 vol.%		
Shaper Materials	Copper				Rubber	
Sz of the Shaper, mm	Ø11 × 1			Ø4 × 1		Ø4 × 1.5
Sr, s^−1^	913	1393	2343	970	1337	1850
Theo.V, m/s	11.10	14.10	20.10	16.36	19.36	22.36
Exp.V, m/s	12.30	15.75	20.55	16.96	19.06	21.15

**Table 6:** Parameters of B_4_C_p_/2024Al composites.

B_4_Cp/2024Al Composites	Density (g/cm^3^)	Young Module (GPa)	Yield Stress (MPa)	Wave Speed (m/s)	Radius (mm)	Thickness (mm)
30 vol%	2.70	184	451	10,240	6	6
70 vol%	2.60	336	917	11,966	6	6

## Data Availability

The data presented in this study are available upon request from the corresponding author.

## References

[1] Ramesh K.T. (2002). Effects of high rates of loading on the deformation behavior and failure mechanisms of hexagonal close-packed metals and alloys. Metall. Mater. Trans. A.

[2] Field J.E., Walley S.M., Proud W.G., Goldrein H.T., Siviour C.R. (2004). Review of experimental techniques for high rate deformation and shock studies. Int. J. Impact Eng..

[3] Kolsky H. (1949). An investigation of the mechanical properties of materials at very high rates of loading. Proc. Phys. Soc. Sect. B.

[4] Gama B.A., Lopatnikov S.L., Gillespie J.W. (2004). Hopkinson bar experimental technique: A critical review. Appl. Mech. Rev..

[5] Yang L., Shim V. (2005). An analysis of stress uniformity in split Hopkinson bar test specimens. Int. J. Impact Eng..

[6] Dao M., Lu L., Shen Y., Suresh S. (2006). Strength, strain-rate sensitivity and ductility of copper with nanoscale twins. Acta Mater..

[7] Lee O.S., You S.S., Chong J.H., Kang H.S. (1998). Dynamic deformation under a modified split Hopkinson pressure bar experiment. KSME Int. J..

[8] Vecchio K.S., Jiang F. (2007). Improved pulse shaping to achieve constant strain rate and stress equilibrium in split-Hopkinson pressure bar testing. Metall. Mater. Trans. A.

[9] Morrow B., Lebensohn R., Trujillo C., Martinez D., Addessio F., Bronkhorst C., Lookman T., Cerreta E. (2016). Characterization and modeling of mechanical behavior of single crystal titanium deformed by split-Hopkinson pressure bar. Int. J. Plast..

[10] Frew D.J., Forrestal M.J., Chen W. (2002). Pulse shaping techniques for testing brittle materials with a split Hopkinson pressure bar. Exp. Mech..

[11] Li X.B., Lok T.S., Zhao J. (2005). Dynamic characteristics of granite subjected to intermediate loading rate. Rock Mech. Rock Eng..

[12] Liang X., Chen G., Lei I.M., Zhang P., Wang Z., Chen X., Lu M., Zhang J., Wang Z., Sun T. (2022). Impact-Resistant Hydrogels by Harnessing 2D Hierarchical Structures. Adv. Mater..

[13] Chao Z., Wang Z., Jiang L., Chen S., Pang B., Zhang R., Du S., Chen G., Zhang Q., Wu G. (2022). Microstructure and mechanical properties of B_4_C/2024Al functionally gradient composites. Mater. Des..

[14] Woo S.-C., Kim T.-W. (2016). High strain-rate failure in carbon/Kevlar hybrid woven composites via a novel SHPB-AE coupled test. Compos. Part B Eng..

[15] Askarinejad S., Johnson J.E., Rahbar N., Troy K.L. (2019). Effects of loading rate on the of mechanical behavior of the femur in falling condition. J. Mech. Behav. Biomed. Mater..

[16] Xu P., Tang L., Zhang Y., Ni P., Liu Z., Jiang Z., Liu Y., Zhou L. (2022). SHPB experimental method for ultra-soft materials in solution environment. Int. J. Impact Eng..

[17] Jiang K., Li J., Kan X., Zhao F., Hou B., Wei Q., Suo T. (2023). Adiabatic shear localization induced by dynamic recrystallization in an FCC high entropy alloy. Int. J. Plast..

[18] Ellwood S., Griffiths L.J., Parry D.J. (1982). Materials testing at high constant strain rates. J. Phys. E Sci. Instrum..

[19] Parry D.J., Walker A.G., Dixon P.R. (1995). Hopkinson bar pulse smoothing. Meas. Sci. Technol..

[20] Naghdabadi R., Ashrafi M.J., Arghavani J. (2012). Experimental and numerical investigation of pulse-shaped split Hopkinson pressure bar test. Mater. Sci. Eng. A.

[21] Duffy J., Campbell J.D., Hawley R.H. (1971). On the use of a torsional split Hopkinson bar to study rate effects in 1100-0 aluminum. J. Appl. Mech..

[22] Li X.B., Lok T.S., Zhao J., Zhao P.J. (2000). Oscillation elimination in the Hopkinson bar apparatus and resultant complete dynamic stress–strain curves for rocks. Int. J. Rock Mech. Min. Sci..

[23] Li X., Zhou Z., Lok T.-S., Hong L., Yin T. (2008). Innovative testing technique of rock subjected to coupled static and dynamic loads. Int. J. Rock Mech. Min. Sci..

[24] Song B., Chen W. (2004). Loading and unloading split Hopkinson pressure bar pulse-shaping techniques for dynamic hysteretic loops. Exp. Mech..

[25] Li W., Xu J. (2009). Impact characterization of basalt fiber reinforced geopolymeric concrete using a 100-mm-diameter split Hopkinson pressure bar. Mater. Sci. Eng. A.

[26] Nemat-Nasser S., Isaacs J.B., Starrett J.E. (1991). Hopkinson techniques for dynamic recovery experiments. Proc. R. Soc. London Ser. A Math. Phys. Sci..

[27] Frew D.J., Forrestal M.J., Chen W. (2001). A split Hopkinson pressure bar technique to determine compressive stress-strain data for rock materials. Exp. Mech..

[28] Frew D.J., Forrestal M.J., Chen W. (2005). Pulse shaping techniques for testing elastic-plastic materials with a split Hopkinson pressure bar. Exp. Mech..

[29] Pang S., Tao W., Liang Y., Liu Y., Huan S. (2019). A modified method of pulse-shaper technique applied in SHPB. Compos. Part B Eng..

[30] Ramírez H., Rubio-Gonzalez C. (2006). Finite-element simulation of wave propagation and dispersion in Hopkinson bar test. Mater. Des..

[31] Song L., Hu S. (2005). Stress uniformity and constant strain rate in SHPB test. Explos. Shock Waves.

[32] Song L., Hu S. Loading designing for constant strain-rate experiment with SHPB. Proceedings of the Fourth Conference on Experimental Techniques in Explosive Mechanics.

[33] Davies E.D.H., Hunter S.C. (1963). The Dynamic Compression Testing of Solids by the Method of the Split Hopkinson Pressure Bar. J. Mech. Phys. Solids.

[34] Lu F., Chen R. (2013). Hopkinson Bar Techniques.

[35] Meyers M.A. (1994). Dynamic Behavior of Materials.

[36] Ravichandran G., Subhash G. (1994). Critical appraisal of limiting strain rates for compression testing of ceramics in a split Hopkinson pressure bar. J. Am. Ceram. Soc..

[37] Wang J., Ma L., Zhao F., Lv B., Gong W., He M., Liu P. (2022). Dynamic strain field for granite specimen under SHPB impact tests based on stress wave propagation. Undergr. Space.

[38] Li Y., Ramesh K., Chin E. (2001). Dynamic characterization of layered and graded structures under impulsive loading. Int. J. Solids Struct..

[39] Johnson G.R., Cook W.H. A constitutive model and data for metals subjected to large strains, high strain rates and high temperatures. Proceedings of the 7th International Symposium on Ballistics.

